# E-Cadherin Promotes Incorporation of Mouse Epiblast Stem Cells into Normal Development

**DOI:** 10.1371/journal.pone.0045220

**Published:** 2012-09-18

**Authors:** Satoshi Ohtsuka, Satomi Nishikawa-Torikai, Hitoshi Niwa

**Affiliations:** 1 Pluripotent Stem Cell Studies, RIKEN Center for Developmental Biology (CDB), Kobe, Japan; 2 CREST (Core Research for Evolutional Science and Technology), Japan Science Technology Agency, Kawaguchi, Japan; National University of Singapore, Singapore

## Abstract

Mouse epiblast stem cells (mEpiSCs) are pluripotent stem cells derived from epiblasts of postimplantation mouse embryos. Their pluripotency is distinct from that of mouse embryonic stem cells (mESCs) in several cell biological criteria. One of the distinctions is that mEpiSCs contribute either not at all or at much lower efficiency to chimeric embryos after blastocyst injection compared to mESCs. However, here we showed that mEpiSCs can be incorporated into normal development after blastocyst injection by forced expression of the E-cadherin transgene for 2 days in culture. Using this strategy, mEpiSCs gave rise to live-born chimeras from 5% of the manipulated blastocysts. There were no obvious signs of reprogramming of mEpiSCs toward the mESC-like state during the 2 days after induction of the E-cadherin transgene, suggesting that mEpiSCs possess latent ability to integrate into the normal developmental process as its origin, epiblasts.

## Introduction

Pluripotent stem cells (PSCs) are defined by their ability to differentiate into the cell types of all three germ layers, *i.e.*, the ectoderm, mesoderm, and endoderm. To date, various types of PSCs from different origin with distinct characters have been reported. It has been proposed that PSCs can be categorized into two major types, naïve and primed PSCs [1]. The former category includes mouse embryonic stem cells (mESCs) [2], [3] and mouse embryonic germ cells, whereas the latter includes mouse epiblast stem cells (mEpiSCs) [4], [5] and human embryonic stem cells [6].

There are several criteria that distinguish naïve and primed PSCs. From the cell biological viewpoint, the most remarkable difference is the ability to contribute to chimeric embryos after blastocyst injection, which is only observed in naïve PSCs. mESCs can contribute to chimeric embryos and form embryos consisting of ESCs when injected into tetraploid blastocysts [7]. In contrast, mEpiSCs barely contribute to chimeric embryos when injected into blastocysts, as Brons *et al*. reported that only 2 chimeras were obtained from 385 injected blastocysts and no germline transmission was observed [5]. However, as mEpiSCs are derived from epiblasts of the postimplantation embryos, they may retain the latent ability to contribute to embryonic development as they originally do *in vivo*. The blastocyst is the orthotopic location for mESCs but an ectopic location for mEpiSCs, which may explain why mEpiSCs were unable to contribute to normal development by blastocyst injection. Indeed, Tesar *et al*. showed that mEpiSCs formed a segregated clump in the blastocyst cavity after injection although mESCs attached to the inner cell mass (ICM) under the same conditions [4]. Therefore, the differences in ability of mESCs and mEpiSCs to contribute to chimeras may be due to the different affinities for ICM attachment rather than their cell biological potential. Orthotopic transplantation of mEpiSCs would be an ideal way to evaluate their ability to contribute to normal development, but it is technically difficult due to the small size of the embryos *in utero* that makes them inaccessible for manipulation.

An alternative is artificial enhancement of mEpiSCs integration into the ICM after blastocyst injection. E-cadherin encoded by *Cadherin 1 (Cdh1)* is responsible for mediating homophilic adhesion of mESCs [8] and its level of expression is higher in mESCs than in mEpiSCs [4], [5]. Therefore, artificial upregulation of E-cadherin in mEpiSCs may accelerate their attachment to the ICM after blastocyst injection and result in efficient generation of chimeric mice. Here, we tested this possibility and succeeded in generating mEpiSC-derived chimeras in a reproducible manner.

## Results

### Establishment of mEpiSCs with Inducible E-cadherin Transgene

To establish EpiSCs in which E-cadherin can be overexpressed in an inducible manner, we introduced a tetracycline-inducible *E-cadherin* expression cassette and tetracycline-dependent activator expression vector into two different EpiSC lines using the piggyBac transposon system [8]. We chose two parental EpiSC lines–female mEpiSCs reported by Tesar *et al.* [4], which is a standard and was designated as PTmEpiSCs in this manuscript, and female mEpiSCs established in our laboratory from 129+ter/SvJcl, designated hereafter as SOmEpiSCs. Use of female EpiSCs allowed us to monitor the X-inactivation status, which is one of the markers for distinguishing between naïve and primed states [4], [5]. We obtained stable cell lines from PTmEpiSCs, designated as EIN3 and EIN6, and from SOmEpiSCs, designated as SvEIN3.4 and SvEIN3.9. These mEpiSCs continued proliferation with no morphological changes after induction of *E-cadherin* transgene expression by addition of doxycycline to the culture medium.

**Figure 1:**
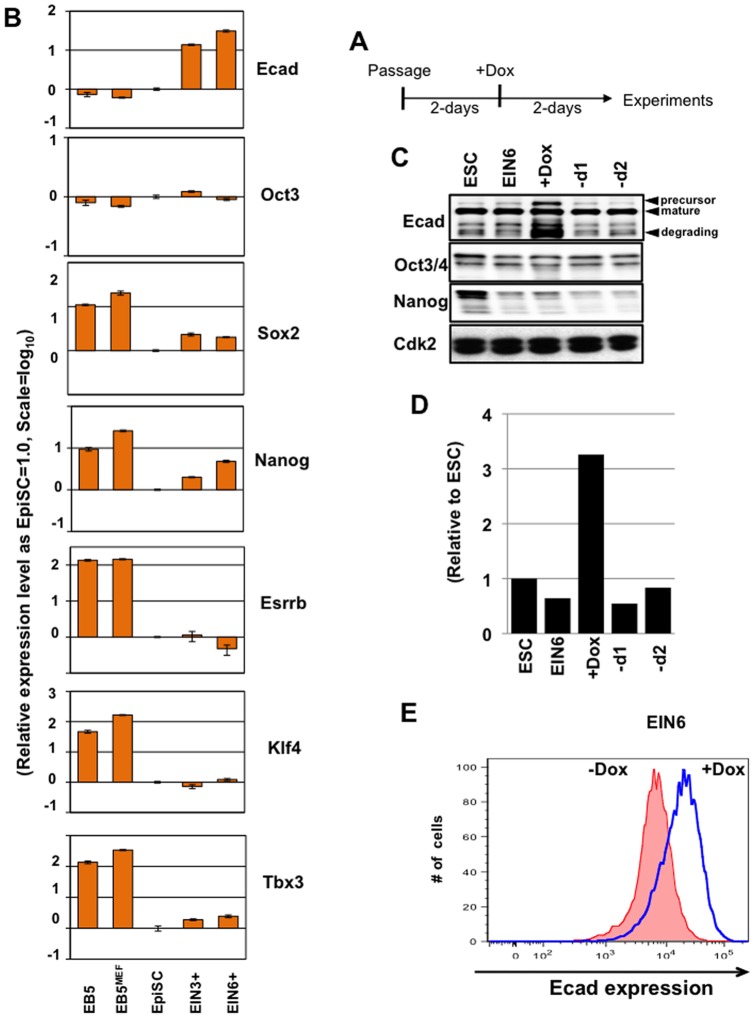
Establishment of mEpiSC lines carrying the doxycycline-inducible *E-cadherin* transgene. A) Time schedule of all experiments with doxycycline (Dox)-inducible expression of E-cadherin in mEpiSCs. mEpiSCs were plated on fibronectin-coated dishes followed by incubation for 24 h, and *E-cadherin* was induced by addition of doxycycline for 2 days. Then, mEpiSCs were dissociated into single-cell suspensions and were subjected to later experiments, such as blastocyst injection, Western blotting, and qPCR analysis. B) qPCR analysis of *E-cadherin* inducible mEpiSCs (EIN3 and EIN6) cultured with or without doxycycline as described in A. The level of expression of each transcript in parental mEpiSCs (PTmEpiSCs) was defined as 1.0. The expression levels in EB5 ES cells cultured with or without the MEF feeder layer (EB5^MEF^ and EB5) are also shown. C) Western blotting analyses for E-cadherin, Oct3/4, and Nanog expression in EB5 ES cells (ESC), EIN6 transgenic mEpiSCs cultured with or without doxycycline for 2 days (EIN6 and +Dox), EIN6 mEpiSCs cultured with doxycycline for 2 days followed by culture without doxycycline for 1 or 2 days (–d1 and –d2). CDK2 is shown as a loading control. D) Quantitative analysis of E-cadherin expression level in Figure 1C. E-cadherin protein levels were normalized to that of Oct3/4 protein in each sample and the level in ESC was defined as 1.0. E) FACS analysis of E-cadherin expression level in EIN6 mEpiSCs cultured with (blue line) or without (red line) doxycycline for 2 days.

First, we applied qPCR analysis to quantify the expression levels of the *E-cadherin* transgene and the endogenous genes associated with pluripotency [9], [10], [11], [12]. The results confirmed the inducible expression of *E-cadherin* in two of the transgenic mEpiSC lines EIN3 and EIN6 derived from PTmEpiSCs (Fig. 1A). Interestingly, we found that the expression levels of the endogenous *E-cadherin* in PTmEpiSCs were slightly higher than those of mESCs cultured with or without feeder cells, suggesting that the ability to integrate into the ICM is not simply correlated with the level of *E-cadherin* transcript. By activation of the *E-cadherin* transgene, *Nanog* expression was slightly upregulated in both lines but the other markers, such as *Oct3/4*, *Klf4*, *Tbx3*, and *Esrrb*, were unaffected. *Klf4*, *Tbx3*, and *Esrrb*, which are known to be specifically expressed in naïve PSCs, showed much higher levels of expression in mESCs than in PTmEpiSCs, as shown in Fig. 1B. It has been reported that these naïve marker genes are upregulated when mEpiSCs are reprogrammed to naïve PSCs by either introduction of transgenes such as *Nanog* [13], [14] or by long-term culture under conditions for naïve PSCs [15]. Therefore, the maintenance of these markers at low levels after induction of the *E-cadherin* transgene in mEpiSCs indicated that upregulation of *E-cadherin* did not induce rapid promotion of reprogramming from the primed to the naïve state. Essentially the same results were obtained with SvEIN3.4, SvEIN3.9, and SOmEpiSCs (data not shown).

Next, we assessed the protein expression levels of E-cadherin in these transgenic EpiSCs by western blotting analysis (Fig. 1C and D). Again, the results indicated that the E-cadherin expression level in PTmEpiSCs was slightly higher than that in feeder-free ESCs. In EIN6 mEpiSCs, E-cadherin protein expression (as the relative amount to Oct3/4 protein) was upregulated to threefold by induction of *E-cadherin* transgene expression with doxycycline, which reverted to the original level within 1 day after withdrawal of doxycycline. Further, by FACS analysis, we confirmed a significant increase in E-cadherin expression level by induction of the E-cadherin transgene with doxycycline (Fig. 1E, comparing –Dox and +Dox). Essentially the same results were obtained for SvEIN3.4, SvEIN3.9, and SOmEpiSCs (data not shown). These findings indicated that there was a significant doxycycline-dependent increase in E-cadherin expression level in these transgenic mEpiSCs.

### Induction of E-cadherin Enhances Integration of mEpiSCs in Normal Development

**Figure 2:**
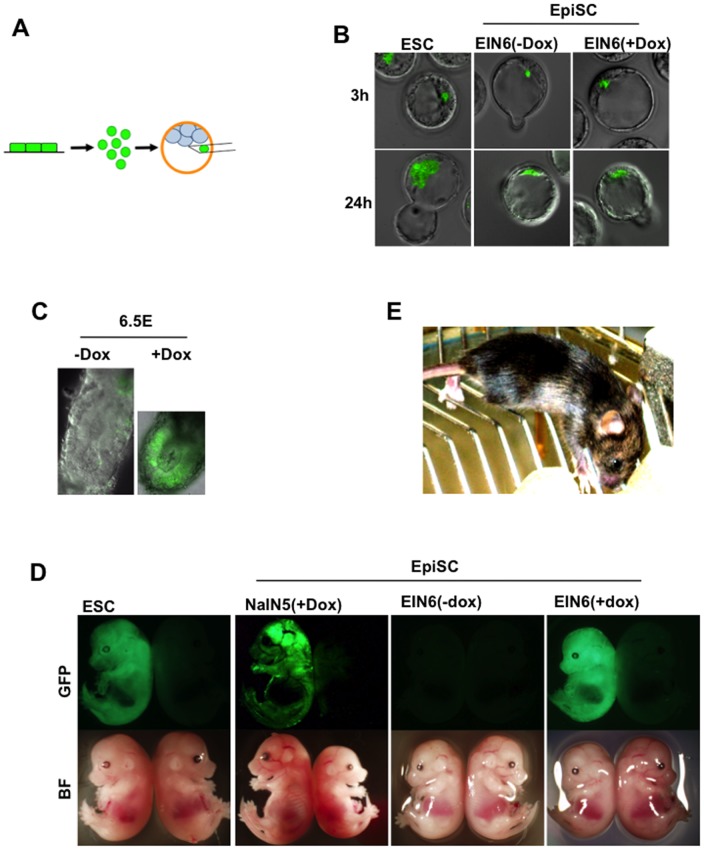
Contribution of mEpiSCs to chimeras after induction of E-cadherin transgene. A) Procedure for single-cell injection. See Materials and Methods for details. B) Localization of the injected mEpiSC at 3 or 24 h after injection into blastocysts. ESCs and EIN6 mEpiSCs cultured with doxycycline (EIN+Dox) and without doxycycline (EIN–Dox) are shown. All cells were labeled by stable transfection of constitutively expressed EGFP transgene for tracing in the embryos. C) Chimeric embryos at E6.5 derived from blastocysts injected with a single EIN6 mEpiSC with EGFP transgene cultured with or without doxycycline before injection. Phase contrast images and fluorescence images of GFP were merged. D) Chimeric embryos at E14.5. NaIN5 and EIN6 mEpiSCs precultured with doxycycline contributed to chimeras at as high efficiency as mESC. E) Live-born offspring derived from EIN3 (+Dox) at 4 weeks age. mEpiSC derived from 129sv with agouti coat color were injected into C57BL6 blastocysts resulting in mice with chimeric coat color.

To examine the effects of increased E-cadherin expression level on incorporation into normal development after blastocyst injection, EIN3 and EIN6 transgenic mEpiSCs were labeled by introduction of the constitutive EGFP expression vector. These EGFP-positive transgenic mEpiSCs were cultured with or without doxycycline for 2 days and dissociated to single cells in the presence of ROCK inhibitor to prevent apoptosis [16]. We attempted to evaluate whether mEpiSC injected with the single mEpiSC injection method was capable of colonizing into developing embryos as efficiently as in the case of ESCs reported previously [17]. When a single mEpiSC was injected into the blastocyst cavity, similar proportions of injected cells were alive in the blastocyst at 24 h after injection in both doxycycline-treated and untreated mEpiSCs, indicating that upregulation of *E-cadherin* did not affect cellular viability (Fig. 2A and B). Interestingly, in both cases, we found a similar proportion of injected cells attached to the ICM at 3 h and incorporated into the ICM at 24 h after injection irrespective of doxycycline treatment (Fig. 2B). The efficiency was comparable to that of mESCs although a previous study indicated that mEpiSCs formed a clump separated from the ICM after blastocyst injection. The differences in observations may be due to the different numbers of cells injected into the blastocyst. In the previous experiment [5], the authors injected multicellular clumps to avoid the apoptosis induced by single-cell dissociation. In contrast, we applied single-cell injection using ROCK inhibitor that prevents apoptosis in dissociated mEpiSCs. Essentially the same results were obtained for SvEIN3.4, SvEIN3.9, and SOmEpiSCs (data not shown). Therefore, under our experimental conditions, no obvious effects of E-cadherin on incorporation into normal development were observed (Fig. 2B).

We examined the contribution of the injected mEpiSCs in postimplantation embryos. Blastocysts injected with a single EIN3 or EIN6 mEpiSC were transferred into the uteri of pseudopregnant female mice and the embryos were dissected at E6.5 (Fig. 2C). Surprisingly, although there was no clear difference between doxycycline-treated and untreated transgenic mEpiSCs in the attachment to ICM in blastocysts, ∼5% of embryos (4 EGFP-positive embryos/63 collected embryos) injected with doxycycline-treated cells carried EGFP-positive cells in epiblasts, whereas no embryos carrying EGFP-positive cells in the epiblast were obtained from blastocysts injected with untreated cells. Instead, we found embryos possessing EGFP-positive cells in the extraembryonic portion, suggesting that mEpiSCs without E-cadherin overexpression were excluded from the epiblasts (Fig. 2C). Therefore, upregulation of E-cadherin promoted incorporation of mEpiSCs into the epiblast along the time course of peri- and postimplantation development.

We examined two transgenic mEpiSCs from each different parental line for their abilities to contribute to chimeric embryos at E13.5 (Fig. 2D and Supplemental Fig. 1). In both cases, ∼5% (1–2 EGFP-positive/40 collected) of blastocysts injected with doxycycline-treated cells gave rise to chimeras (Table 1 and Supplemental Table 1) in which EGFP-positive E-cadherin-overexpressing mEpiSCs made a large contribution (Fig. 2D). None of the blastocysts injected with untreated cells resulted in chimeric embryos, confirming the inability of wild-type mEpiSCs to form chimeras. The efficiency to give rise to chimeric embryos was not altered when the transgenic mEpiSCs were cultured with doxycycline for 10 passages prior to injection (Table 1), suggesting that the promotion of chimera contribution of mEpiSCs by E-cadherin was dependent on its short-term effect and was not a progressive event such as reprogramming. Finally, we obtained live-born chimeric mice from doxycycline-treated mEpiSCs of all 4 transgenic mEpiSCs (Fig. 2E), indicating normal pluripotency of mEpiSCs in this context, although none showed germline transmission.

**Table 1:** Efficiency of chimera generation from EpiSCs with (+Dox) or without (–Dox) E-cadherin induction.

Parental Cell Line		24 h survival rate	GFP(+)/total embryos	# of injected
PT mEpiSC	EIN3	61.5%	0/53	112
	EIN3 (+Dox)	70.5%	1/26	112
	EIN3 (+Dox)p10	n.d.	1/28	80
	EIN6	69.2%	0/44	112
	EIN6 (+Dox)	81.8%	1/25	96
	EIN6 (+Dox)p10	n.d.	1/18	66
ESC	ESC	91%	11/47	140

To evaluate 24-h survival rate of injected EpiSCs, injected blastocysts were randomly picked followed by microscopic observation of EGFP-positive EpiSCs. Remaining injected blastocysts were transferred into the uterus of a pseudopregnant ICR female. At 13.5E, mice were sacrificed to collect embryos to evaluate chimerism of the injected EpiSC. ESCs were used as a control to determine chimerism.

### Induction of E-cadherin does not Trigger Obvious Reprogramming

**Figure 3:**
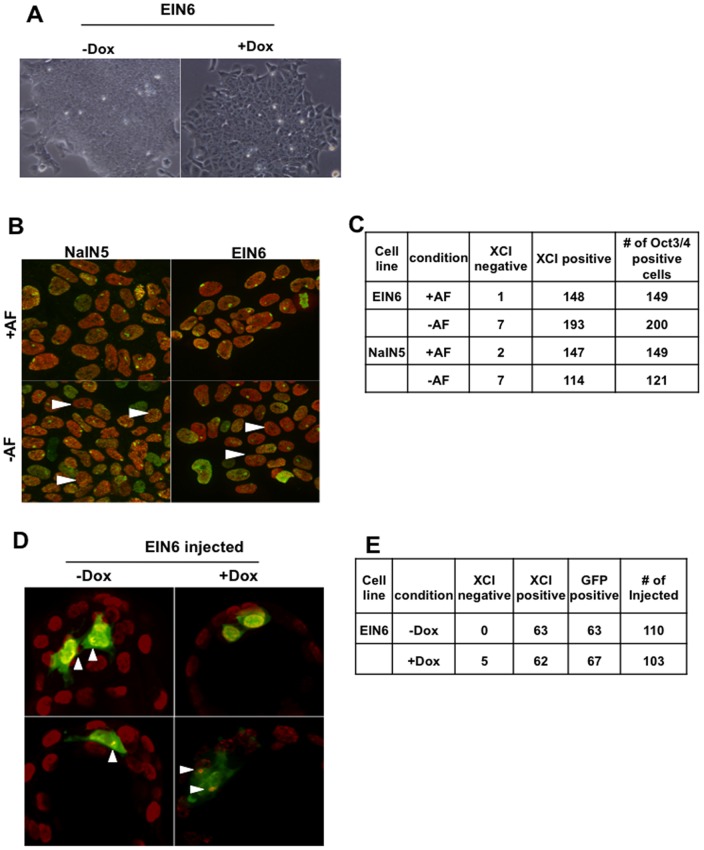
Effects of E-cadherin transgene expression on X-inactivation status. A) Morphology of EpiSCs with or without induction of E-cadherin upon addition of doxycycline (-Dox and +Dox). B) The X-inactivation status of Nanog-induced (NaIN5) or E-cadherin-induced (EIN6) EpiSCs. The Nanog or E-cadherin transgene was induced with doxycycline for 2 days with or without Activin and Fgf2 (+AF or –AF) and stained with H3K27me3 (shown in green) and Oct3/4 (shown in red). White arrowheads indicate inactive X chromosome (XCI)-negative cells. C) Measurement of the ratio of XCI-negative and -positive cells in Figure 3B. Only Oct3/4-positive cells were counted in each case. Note that NaIN and EIN cells maintained 100% XCI in EpiSC medium without doxycycline (data not shown). D) E-cadherin induced EpiSCs lost XCI in injected blastocysts cultured for 36 h. EpiSCs induced by E-cadherin for 2 days were injected into blastocysts with the single-injection protocol. The embryos were then cultured for 36 h followed by immunostaining with H3K27me3 antibody to estimate reprogramming events in developing embryos. EGFP-positive cells (green) and H3K27me3-positive (red) cells were counted in injected embryos. E) Measurement of the ratio of XCI-positive and negative cells in Figure 3D. Only GFP positive cell were counted (p<0.05 between each pair).

It has been reported that mEpiSCs can convert to naïve PSCs and acquire the ability to form chimeras [13], and E-cadherin is a factor that can cooperate with transcription factors to promote reprogramming of somatic cells to naïve PSCs [18]. Therefore, promotion of reprogramming to naïve PSCs is one possible mechanism for the effect of *E-cadherin* overexpression on chimera formation of mEpiSCs. Several criteria have been proposed to distinguish between naïve and primed PSCs. *Klf4*, *Tbx3*, and *Esrrb* are marker genes for naïve PSCs [1] but we found that *E-cadherin* overexpression did not activate these genes (Fig. 1B). Naïve PSCs are characterized by dome-like compact colonies with multilayered cells but E-cadherin-overexpressing mEpiSCs maintained the flat colony morphology characteristic of primed PSCs (Fig. 3A). Here, we examined the activation status of the X chromosome in female mEpiSCs as a marker of primed PSCs as X-inactivation in females occurs in the transition from naïve to primed PSCs [19]. It was reported previously that 99% of female mEpiSCs established from postimplantation epiblasts carried the large foci of histone H3 lysine 27 trimethylation (H3K27me3), which is a hallmark of the inactive X chromosome [14], [20]. We found that 100% of the EIN6 mEpiSCs carrying the inducible *E-cadherin* transgene possessed inactive X chromosome foci in culture without doxycycline. A number of cells without the foci appeared 2 days after induction of the *E-cadherin* transgene by doxycycline, but they represented less than 1% of the cells counted (Fig. 3A and B). Essentially the same results were obtained for EIN3 mEpiSCs (data not shown). *Nanog* is a known inducer of reprogramming of mEpiSCs to naïve PSCs [14], but the effect of *Nanog* overexpression induced by doxycycline on removal of X-inactivation foci was also faint at this time (<2%), suggesting that obvious reprogramming was not induced within 2 days of doxycycline treatment under mEpiSC culture conditions.

As no obvious effect of *E-cadherin* overexpression was observed when the transgenic mEpiSCs were injected into blastocysts, we examined the X-inactivation state in transgenic mEpiSCs in this context. In the case of untreated EIN6 mEpiSCs, all EGFP-positive cells in blastocysts at 24 h after injection maintained the H3K27me3 foci. In contrast, 14% of EGFP-positive cells derived from doxycycline-treated transgenic EIN6 EpiSCs lost the foci, suggesting their transition to the naïve state. This was slightly higher than the proportion losing the inactive X chromosome foci with induction of the *E-cadherin* transgene for 2 days in the absence of fibroblast growth factor (Fgf)-2 and Activin, which was about 4% (Fig. 3B–AF). It was reported that Fgf2 and Activin play inhibitory roles in reprogramming induced by *Nanog*, so leaving the mEpiSCs in the blastocyst without Fgf2 and Activin may have some positive effect on promoting reprogramming events. However, it was technically impossible to confirm the link between the loss of X-inactivation and integration into chimeric embryos (Fig. 3C).

## Discussion

mEpiSCs are primed PSCs derived from postimplantation mouse embryos. They continue to show self-renewal in medium containing Fgf-2 and Activin [4], [5]. As it was reported that similar primed stem cells can be established from the ICM of blastocysts when cultured in medium containing Fgf2 and Activin, the state of PSCs is primarily determined by the culture conditions rather than their origin. In non-rodent animals, ESCs were established from ICM in medium containing Fgf2 and Activin [21], [22]; these were primed PSCs that did not contribute to chimeric animals after blastocyst injection, although there is interest in production of chimeric animals from the viewpoint of basic science as well as industrial applications. Here, we demonstrated that E-cadherin overexpression promotes the contribution of mEpiSCs to chimeric mouse embryos, which may also be applicable for non-rodent animals.

mEpiSCs were originally established from E5.5–E5.75 mouse epiblasts. However, they may mimic the characteristics of later-stage epiblasts. Recently, Hayashi *et al.* reported that mESC-derived epiblast-like cells can differentiate to primordial germ cells *in vitro*, but mEpiSCs cannot, and they found that the global gene expression profile of mEpiSCs is different from that of E5.75 epiblasts [23]. Therefore, in medium containing Fgf2 and Activin, primed PSCs may be stabilized at this developmental stage, equivalent to the late epiblast after segregation to primordial germ cells, irrespective of their origin. This may explain why we failed to observe germline transmission in our mEpiSC-derived chimeras although the contribution was not low.

The ability to form chimeras by incorporation into the normal developmental course after blastocyst injection is regarded as one of the hallmarks of naïve PSCs [1]. Indeed, we found no contribution of mEpiSCs to chimeric embryos without *E-cadherin* overexpression (Table 1 and Supplemental Table 1). However, activation of the *E-cadherin* transgene for 2 days prior to injection allowed these cells to incorporate into epiblasts at E6.5 (Fig. 2C). Within the 2 days of culture with doxycycline *in vitro*, we observed no signs of obvious reprogramming of mEpiSCs to naïve PSCs. Previous reports indicated that reprogramming mediated by the overexpression of exogenous factors, such as *Nanog*, took 10 days [14], and that promoted by the culture conditions for mESCs required several weeks [15]. These observations suggested that full reprogramming may not occur in such a short period. Moreover, the frequency of acquisition of the ability to form chimeras by the cells was much higher than the reprogramming efficiency reported previously [14]. We showed that about 5% of blastocysts carrying single transgenic mEpiSCs gave rise to viable chimeras. As 23% of blastocysts carrying single mESCs resulted in generation of chimeras (Table 1), the actual rate of establishing the ability to form chimeras in mEpiSCs by E-cadherin should have been higher than 5%, and could reach 20%. If reprogramming occurred with such high efficiency, it would be reflected in the upregulation of naïve markers and increase in cells without inactive X chromosomes, but none of these were actually observed. These data strongly supported the hypothesis that E-cadherin promotes integration of mEpiSCs into epiblasts without reprogramming and mEpiSCs have latent ability to contribute to embryonic development as their origin, epiblasts, do in the normal developmental context.

However, the possible reprogramming event was not completely excluded because we found inactive X chromosome loss in a significant proportion of the transgenic mEpiSCs after injection into blastocysts (Fig. 3C and D). These observations suggest that the interaction of mEpiSCs with ICM promotes rapid reprogramming, which was enhanced by *E-cadherin* overexpression. To confirm whether reprogramming to the naïve state is required for mEpiSCs to contribute to chimeras, it will be necessary to inject them into the orthotopic position, postimplantation embryos, and determine whether they incorporate into normal development. These will be very challenging experiments due to the technical difficulty, but such studies are essential to expand our knowledge regarding the characteristics of naïve and primed PSCs.

## Materials and Methods

### Plasmid Construction

*E-cadherin* and *Nanog* cDNAs were amplified by PCR using the following primers: *E-cadherin* 5′-tttgtcgacgggtccgccatgggagcccggt-3′ and 5′-tttgcggccgcctagtcgtcctcaccaccgccgt-3′; *Nanog* 5′-tttCTCGAGGCCGACTGAGTGTGGGTCTTCCTGGTCC-3′ and 5′-tttGCGGCCGCCTCTTTCACCTGGTGGAGTCACAG-3′ (restriction sites are underlined). The cDNAs were cloned into pBluescript and were confirmed by sequencing. These cloned fragments were digested with *Sal*I and *Not*I (for *E-cadherin*) or *Xho*I and *Not*I (for *Nanog*), and then ligated into *Xho*I–*Not*I-cleaved pPB-hCMV*1-pA vector.

### Derivation and Culture of mEpiSC Lines

The mEpiSC lines were established following the procedure of Tesar *et al.* [4]. Briefly, female and male 129+ter/SvJcl mice (purchased from Clea Japan Inc.) were mated, and 6.5E embryos were collected from the uterus. The endoderm layer of the embryos was peeled off manually with glass needles, and transferred into mEpiSC medium consisting of DMEM-F12, 20% knock-out serum replacement, 1 mM sodium pyruvate, 1x non-essential amino acids (Nacalai Tesque), 10^−4^ M 2-ME, 10 ng/mL activin A (Wako Pure Chemical Industries, Ltd.), and 10 ng/mL human recombinant FGF2 (Peprotech) on mitomycin C-treated mouse embryonic fibroblasts. Growing colonies were typically observed within a few days with differentiated cells. To purify stem cell colonies, differentiated cells surrounding the stem cells were discarded by manually picking under a microscope (until passage 5). Cells were routinely passaged on fibronectin (BD Biosciences)-coated dishes every 3 days using accutase (Sigma) or 1 mg/mL collagenase type-IV (Gibco) dissolved in DMEM-F12 at a concentration of 10 mg/mL as a stock solution. Thereafter mEpiSCs were usually passaged with enzyme treatment as described above. After several passages (>5 passages on MEF feeder layer), mEpiSCs were usually stably self-renewing. mEpiSCs were adapted and maintained under feeder-free conditions as described previously [13]. All experiments described in this manuscript were performed under feeder-free culture conditions.

### Chimera Formation Assay

One single dissociated mEpiSC in the presence of ROCK inhibitor (10 µM Y-27632, Wako Pure Chemical Industries, Ltd.) was carefully injected into a C57BL6 blastocyst by microinjection, which was then transferred into the uterus of a pseudopregnant female ICR mouse. Embryos were collected at 13.5 dpc to evaluate chimera contribution ability of mEpiSC. Collected embryos were analyzed by fluorescence microscopy. Live-born chimeras were delivered naturally or by cesarean section at the day of birth.

### Transfection

*E-cadherin* or *Nanog* inducibly expressing cell lines (EIN3, 6, and NaIN5, respectively) were established by transfection with LipofectAMINE 2000 (Invitrogen) according to the manufacturer’s instructions. mEpiSCs were dissociated with accutase (Invitrogen), and then incubated with medium/DNA/LipofectAMINE 2000 mixture for 3 h, then changed to fresh medium. After 3 days, cells were split into a 1∶10 ratio in fibronectin-coated dishes and maintained until G418-resistant colonies were grown. Transgene induction levels in clones were confirmed using qPCR analysis comparing plus and minus induction for 2 days. The plasmids used were as follows: NaIN cell line, pCAGGS-PBase, pPBCAG-EGFPiZ (iZ; IRES-Zeocin resistance gene cassette), pPBCAG-rtTAIN (IN; IRES-Neomycin resistance cassette), and pPBCMV*1-Nanog; EIN cell line, pCAGGS-PBase, pPBCAG-EGFPiZ, pPBCAG-rtTAIN, and pPBCMV*1-E-cadherin.

### Immunostaining and FACS Analysis

Immunostaining analysis of cells was performed as described previously [9]. Cells were cultured for the indicated times in each experiment on fibronectin-coated ibidi-treated chamber slides (Nippon Genetics Co., Ltd.), fixed with 4% paraformaldehyde, and stained with the following primary antibodies: anti-E-cadherin (kindly provided by Dr. Masatoshi Takeichi), H3K27me3 antibody (1∶2000 dilution, #07-449; Millipore Corporation), and anti-Oct3/4 (1∶2000 dilution, C-10; Santa Cruz Biotechnology Inc.). All secondary antibodies used were Alexa Fluor highly cross-adsorbed (Molecular Probes). The slides were then imaged using a confocal microscope (Leica). For FACS analysis of cells, single-cell suspensions were stained with anti-E-cadherin antibody and Alexa Fluor-labeled secondary antibody. The stained cells were analyzed by FACSAria (BD Biosciences) and the data were analyzed with FlowJo software.

### Quantitative PCR

To quantify the levels of transcripts, cDNAs were synthesized from 1 mg of total RNA using ReverTra Ace (Toyobo), and evaluated by quantitative PCR using a BioRad CFX384 Real-time system and the primers as described previously [9] except primers for E-cadherin, which were as follows: 5′-AAC AAC TGC ATG AAG GCG GGA ATC-3′ and 5′-CCT GTG CAG CTG GCT CAA ATC AAA-3′. All samples were tested in triplicate, and the results of each were normalized relative to GAPDH expression. The mean relative amounts of each transcript were calculated.

### Animal Ethics Statement

All animal experiments conformed to our Guidelines for the Care and Use of Laboratory Animals and were approved by the Institutional Committee for Laboratory Animal Experimentation (RIKEN Kobe Institute).

## Supporting Information

Figure S1 **13.5E chimeric embryos derived from SvEIN3.4 EpiSCs possessing inducible E-cadherin transgene used in** **Figure 3** **.**
(TIF)

Table S1 **Efficiency of chimera generation from EpiSCs with (+Dox) or without (–Dox) E-cadherin induction in SvEIN3.4 and SvEIN3.9 EpiSCs.**
(DOCX)
