# Supplementary figures and images for: E-Cadherin Promotes Incorporation of Mouse Epiblast Stem Cells into Normal Development
Source: PLoS One. 2012 Sep 18;7(9):e45220. doi: 10.1371/journal.pone.0045220 (PMC3445497; doi:10.1371/journal.pone.0045220)

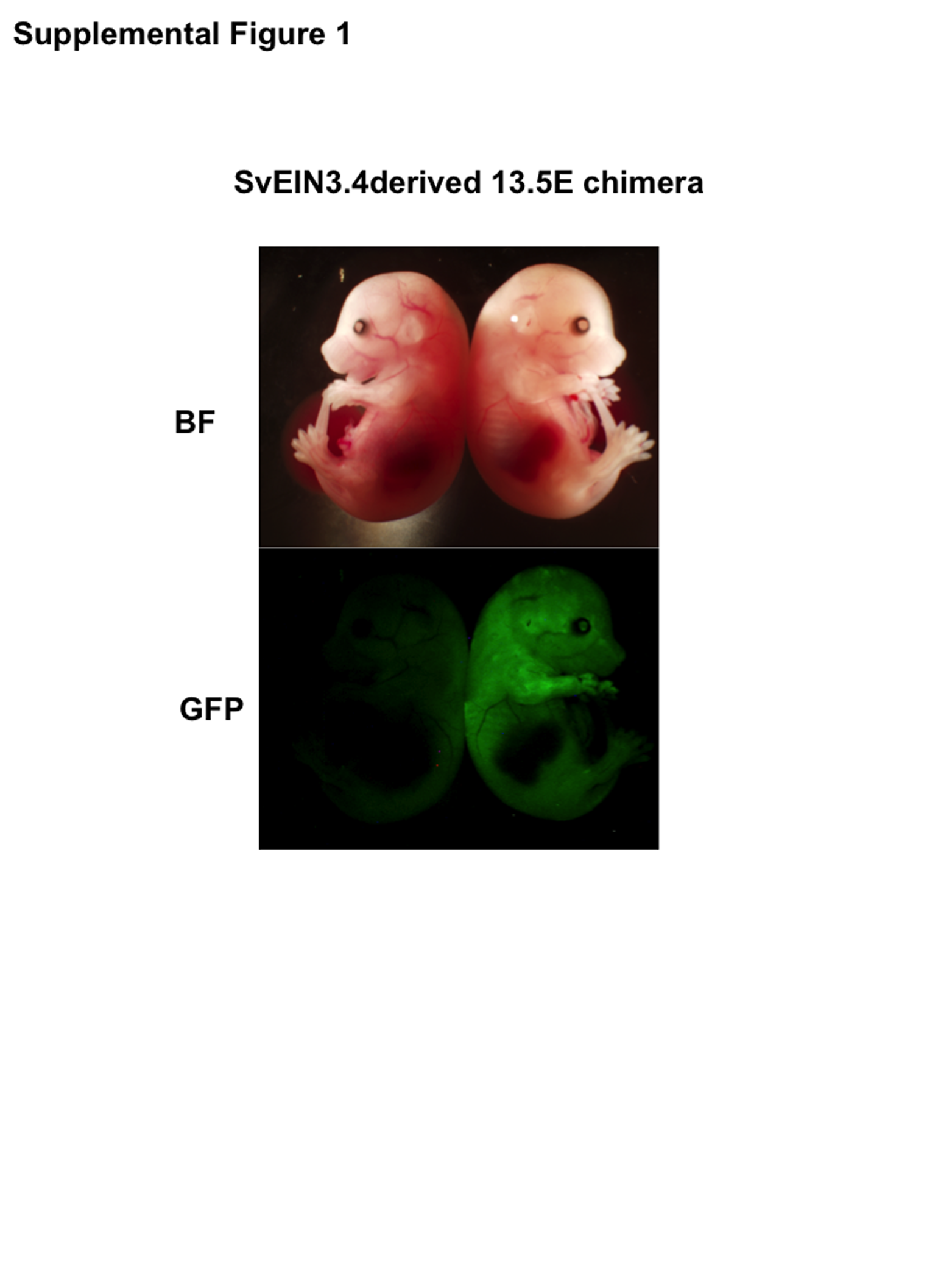

Supplement: Figure S1 — 13.5E chimeric embryos derived from SvEIN3.4 EpiSCs possessing inducible E-cadherin transgene used in Figure 3 . (TIF) [file pone.0045220.s001.tif]
