# Supplementary material for: E-Cadherin Promotes Incorporation of Mouse Epiblast Stem Cells into Normal Development
Source: PLoS One. 2012 Sep 18;7(9):e45220. doi: 10.1371/journal.pone.0045220 (PMC3445497; doi:10.1371/journal.pone.0045220)
Supplement: Table S1 — Efficiency of chimera generation from EpiSCs with (+Dox) or without (–Dox) E-cadherin induction in SvEIN3.4 and SvEIN3.9 EpiSCs. (DOCX) [file pone.0045220.s002.docx]

| **Cell Line** | **condition** | **GFP(+) / collected / Injected** |
| --- | --- | --- |
| **SvEIN3.4** | **-Dox** | **0 / 30 / 128** |
|  | **+Dox** | **2 / 59 / 144** |
|  |  |  |
| **SvEIN3.9** | **-Dox** | **0 / 22/ 120** |
|  | **+Dox** | **1 / 33 / 120** |

Supplemental Table 1

Different parental EpiSC-derived chimera embryos
